# The Incidence of Stroke in Indigenous Populations of Countries With a Very High Human Development Index: A Systematic Review Protocol

**DOI:** 10.3389/fneur.2021.661570

**Published:** 2021-04-22

**Authors:** Anna H. Balabanski, Angela Dos Santos, John A. Woods, Amanda G. Thrift, Timothy J. Kleinig, Astrid Suchy-Dicey, Susanna Ragnhild Siri, Bernadette Boden-Albala, Rita Krishnamurthi, Valery L. Feigin, Dedra Buchwald, Annemarei Ranta, Christina S. Mienna, Carol Zavaleta, Leonid Churilov, Luke Burchill, Deborah Zion, W. T. Longstreth, David L. Tirschwell, Sonia Anand, Mark W. Parsons, Alex Brown, Donald K. Warne, Matire Harwood, Judith M. Katzenellenbogen

**Affiliations:** ^1^Department of Medicine, School of Clinical Sciences at Monash Health, Monash Universit, Melbourne, VIC, Australia; ^2^Department of Medicine and Neurology, Melbourne Brain Centre at Royal Melbourne, Melbourne, VIC, Australia; ^3^Western Australian Centre for Rural Health, School of Population and Global Health, University of Western Australia, Perth, WA, Australia; ^4^Department of Neurology, Royal Adelaide Hospital, Adelaide, SA, Australia; ^5^Elson S. Floyd College of Medicine, Washington State University, Spokane, WA, United States; ^6^Department of Community Medicine, Faculty of Health Sciences, Centre for Sami Health Research, UiT the Arctic University of Norway, Tromso, Norway; ^7^Department of Population Health and Disease Prevention, Department of Epidemiology, University of California, Irvine, Irvine, CA, United States; ^8^National Institute for Stroke and Applied Neurosciences, Auckland University of Technology, Auckland, New Zealand; ^9^Institute for Research and Education to Advance Community Health, Washington State University, Seattle, WA, United States; ^10^Department of Medicine, University of Otago, Wellington, New Zealand; ^11^Faculty of Medicine, Umeå University, Umeå, Sweden; ^12^Facultad de Salud Pública y Administración, Universidad Peruana Cayetano Heredia, Lima, Peru; ^13^Department of Medicine, Royal Melbourne Hospital, University of Melbourne, Melbourne, VIC, Australia; ^14^Human Research Ethics Committee, Victoria University, Melbourne, VIC, Australia; ^15^Departments of Neurology and Epidemiology, University of Washington, Seattle, WA, United States; ^16^Department of Medicine, Faculty of Health Sciences, McMaster University, Hamilton, ON, Canada; ^17^University of New South Wales (UNSW) South Western Sydney Clinical School, Liverpool, NSW, Australia; ^18^Wardliparingga Aboriginal Health Equity Theme, South Australian Health and Medical Research Institute, Adelaide, SA, Australia; ^19^School of Medicine and Health Sciences, University of North Dakota, Grand Forks, ND, United States; ^20^Te Kupenga Hauora Māori, Faculty of Medical and Health Sciences, University of Auckland, Auckland, New Zealand; ^21^School of Population and Global Health, University of Western Australia, Perth, WA, Australia

**Keywords:** epidemiology, incidence, population, stroke, health, aboriginal, indigenous

## Abstract

**Background and Aims:** Despite known Indigenous health and socioeconomic disadvantage in countries with a Very High Human Development Index, data on the incidence of stroke in these populations are sparse. With oversight from an Indigenous Advisory Board, we will undertake a systematic review of the incidence of stroke in Indigenous populations of developed countries or regions, with comparisons between Indigenous and non-Indigenous populations of the same region, though not between different Indigenous populations.

**Methods:** Using PubMed, OVID-EMBASE, and Global Health databases, we will examine population-based incidence studies of stroke in Indigenous adult populations of developed countries published 1990-current, without language restriction. Non-peer-reviewed sources, studies including <10 Indigenous People, or with insufficient data to determine incidence, will be excluded. Two reviewers will independently validate the search strategies, screen titles and abstracts, and record reasons for rejection. Relevant articles will undergo full-text screening, with standard data extracted for all studies included. Quality assessment will include Sudlow and Warlow's criteria for population-based stroke incidence studies, the Newcastle-Ottawa Scale for risk of bias, and the CONSIDER checklist for Indigenous research.

**Results:** Primary outcomes include crude, age-specific and/or age-standardized incidence of stroke. Secondary outcomes include overall stroke rates, incidence rate ratio and case-fatality. Results will be synthesized in figures and tables, describing data sources, populations, methodology, and findings. Within-population meta-analysis will be performed if, and where, methodologically sound and comparable studies allow this.

**Conclusion:** We will undertake the first systematic review assessing disparities in stroke incidence in Indigenous populations of developed countries. Data outputs will be disseminated to relevant Indigenous stakeholders to inform public health and policy research.

## Introduction

### Rationale

This study has been undertaken to investigate the incidence of a single health condition—stroke—locating the research in the context of Indigenous health more broadly. The authors intend to undertake the research in a way that is culturally responsive and respectful of the included populations, while also promoting understanding within the scientific and general communities to broadly support improved primary prevention of stroke.

### The Health of Indigenous Peoples

There are an estimated 370 million Indigenous people living across 90 countries worldwide (1). Indigenous and tribal populations (hereafter referred to as Indigenous Peoples, though we respectfully acknowledge that this may not be the preferred term for all peoples) are the traditional custodians of many ecologically and economically diverse regions around the globe (1, 2). There is great variance in historical, cultural, epistemological, socioeconomic and environmental factors within and between these populations, thereby, greatly enriching the cultural and linguistic diversity of the world today (1, 3).

The historical consequences of colonization of Indigenous Peoples across the globe have left a legacy of economic and health disparity, typically disadvantaging Indigenous populations (4–8). Many Indigenous Peoples have had lands misappropriated, and their freedom to practice their traditional cultures and lifestyles curtailed, resulting in a complex network of disadvantage (4–6, 9, 10). While income disparity and disadvantage are not limited to Indigenous populations, in most countries in the world racism has been particularly virulent toward Indigenous Peoples (4), and in many cases continues (5–7, 11). While institutionally and scientifically sanctioned racist views are now widely discredited, the legacy of these views and policies persists in many ways (6).

In 2007, the United Nations Declaration on the Rights of Indigenous Peoples (12) affirmed the principles of Indigenous Peoples' right to self-determination, including their rights to freely determine their political status and to pursue socioeconomic, health and cultural development (12). Despite these recent gains, Indigenous populations typically (though not uniformly) experience substantial disadvantages in socioeconomic status and health outcomes relative to the general population in their countries (4, 13–19). This is especially true of countries with a Very High Human Development Index (HDI) where, on average, the general population enjoy a high quality of life and life expectancy, yet these indicators often do not extend to their Indigenous, colonized populations (3, 9, 10, 20).

Our review will be focused specifically on Indigenous populations living in developed countries, where there is access to advanced technological infrastructure and financial resources to manage and prevent stroke. In theory, disparities present between Indigenous and non-Indigenous populations in these countries are less likely to reflect nationally reduced access to resources. By identifying regions where there is relatively less disadvantage, or who have effectively improved health outcomes over time, we can examine which approaches and interventions have been most effective in achieving better health outcomes in reducing the incidence of stroke.

### Role of Conventional Health Research in Perpetuating Inequalities in Indigenous Health

#### Conceptualization of Health

Historically, health research involving Indigenous populations has not always translated into improved health outcomes for Indigenous Peoples (21–23). Such research may have been designed and conducted applying Western notions of health to the Indigenous context, often without regard for the priorities of the Indigenous population in question (22, 24, 25). Indigenous Peoples' concept of health and often encompasses a more holistic view, incorporating physical, emotional, mental and spiritual health, along with a connection to family, community and their land (3, 9, 10, 19, 26–28).

#### Data Quality and Ownership

The incremental gains in health and well-being resulting from a greater evidence base and improved technology have often been smaller for some Indigenous populations, relative to their corresponding non-Indigenous population (3, 21, 29, 30). These limited gains have partly been attributed to a shortage of solutions-based research designs, such as studies developing and evaluating the efficacy of culturally responsive health interventions (20, 21). Studies lacking rigorous design have led to poor data quality, providing a poor foundation upon which to build change (20, 21). Furthermore, much research has not adhered to data sovereignty principles, including Indigenous Peoples' control over the collection and use of their own data (31). Evidence-based actions are urgently required to address the health disparities (3, 9, 13, 32).

#### Community Participation in Research Processes

Health research in Indigenous populations has often been conducted in an exploitative manner, sometimes resulting in significant harm to these populations (21, 24, 29, 33–36). Health inequity cannot be fully understood without considering the social determinants of health including historic and persistent systemic and institutional racism (4, 8, 9, 26, 37–39). Yet these inequalities have frequently been attributed to “poorer behavior” (40) of Indigenous groups. This depiction of Indigenous health in terms of “absence, lack, or failure” within scientific and public communication, policy and practices, reflects the typical deficit discourse (41). Such discourse promotes inaccurate, harmful perceptions of existing health disparities, perpetuating individual and systemic racism, and promoting the continued imbalance in power between Indigenous and non-Indigenous Peoples (40). Consequently, Indigenous People of Australia (21, 23) and other Indigenous Peoples (42) have questioned the utility of health research in promoting meaningful improvements in their health and well-being.

### Health Research to Support Improvement of Indigenous Health Outcomes

Indigenous health research and interventions should be shaped around Indigenous Peoples' own notion of health, and conducted in a culturally responsive manner through Indigenous governance, community consultation, engagement and partnership (9, 26, 43, 44). Through examining national and international guidelines on Indigenous health research, Huria et al. developed the consolidated criteria for strengthening reporting of health research involving Indigenous Peoples (CONSIDER statement) (22). This statement identifies eight research domains (governance; relationships; prioritization; methodologies; participation; capacity; analysis and findings; and dissemination) critical to designing and conducting research which supports improvements in Indigenous health outcomes (22). We utilized these criteria to inform the design of this protocol.

### Indigenous Health and Stroke

**Figure 1:**
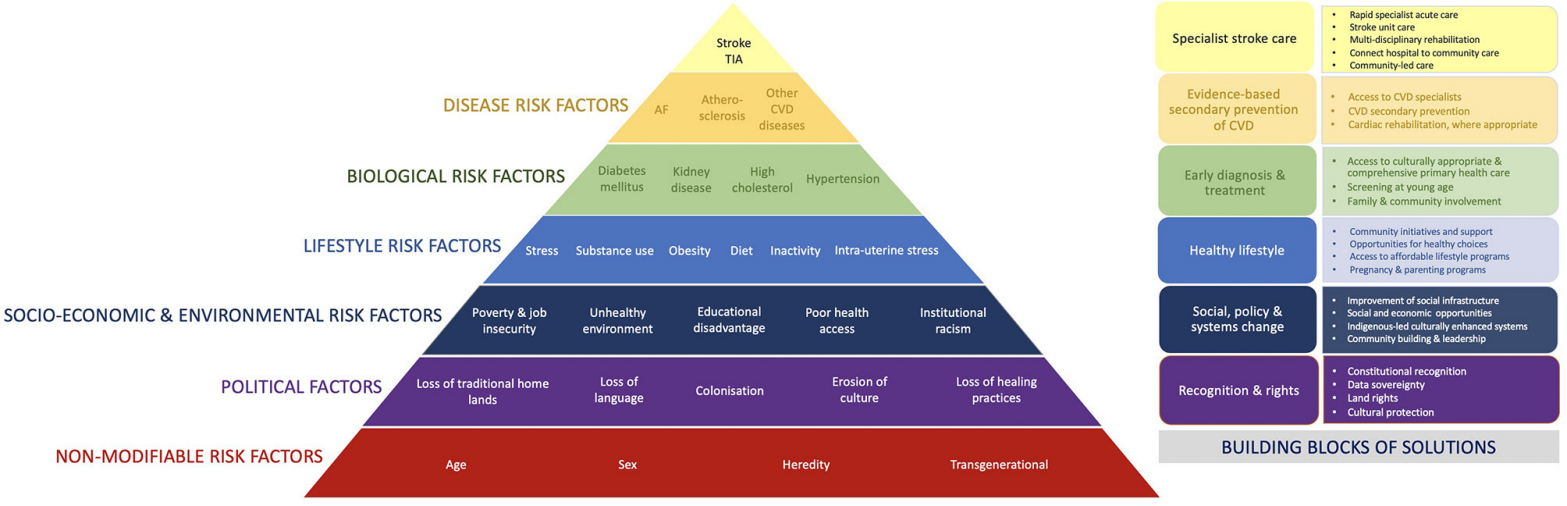
Pyramid of stroke in Indigenous populations.

Cardiovascular diseases, including stroke, contribute significantly, and often disproportionally greater, to the overall burden of disease among many Indigenous populations (4, 14, 17, 45, 46). Stroke is particularly significant, being the second most common cause of death and third greatest cause of disability worldwide, creating substantial burden to individuals, families, communities, and health systems (47). The incidence and outcomes of stroke are the consequence of a complex interplay between numerous individual and environmental factors (Figure 1). The social determinants of health, particularly significant for many Indigenous populations, may predispose to stroke. This appears to be generally supported by a disproportionate burden of stroke affecting these populations, often occurring at younger ages than in non-Indigenous populations (48–57). However, published data on the epidemiology of stroke in Indigenous populations are few and, to date, no reviews have been undertaken to systematically investigate the incidence of stroke in the Indigenous populations globally. Such information is urgently needed to underpin advocacy for evidence-based, effective health interventions (20).

The purpose of this study is to undertake a systematic review of peer-reviewed data to provide a greater understanding of the incidence of stroke (including ischemic stroke, spontaneous intracerebral hemorrhage and non-traumatic subarachnoid hemorrhage, and excluding transient ischemic attacks) among Indigenous Peoples in developed countries. This information will be used to identify gaps in knowledge to guide future research, and to inform healthcare policy development and service reform to reduce inequities and improve stroke health outcomes following stroke in Indigenous populations.

## Objectives

To synthesize published data on the incidence of stroke and its pathological types in Indigenous populations of developed countries/regions, with comparisons (where available) made between Indigenous and non-Indigenous populations, and within Indigenous populations including by sex, age groups and location (i.e., urban, rural, and remote populations), where stratified data are provided (Table 1).

**Table 1:** PICO framework.

Population	Indigenous and non-Indigenous adult populations of developed countries
Intervention	Not applicable.
Comparators	Indigenous vs. non-Indigenous populations of specific regions, stratified by:
	1. Sex 2. Age group 3. Urban and rural/remote location *Note, direct comparisons between different Indigenous populations will not be undertaken*.
Outcomes	Primary: Crude, age-specific or age-standardized incidence (first-ever-in-a-lifetime) rates of stroke and their rate ratios; Secondary: attack rates (all cases) of stroke; case-fatality

### Indigenous Governance

This research is being undertaken with oversight from an Indigenous Advisory Board (co-authors AD, CM, MH, LB, AB, DW) comprised of Indigenous researchers from different high-income countries. This is to ensure all aspects of the research, including the design, conduct, interpretation, reporting and publication, are undertaken in a culturally appropriate manner, and for the benefit of Indigenous Peoples. We sought to engage these Indigenous researchers in order to be guided by their local knowledge, insight and perspectives. We have developed our research aims in accordance with the priorities identified by relevant Indigenous governing bodies. We further developed the protocol to adhere to, and honor, Indigenous ethical guidelines and processes. We acknowledge that all data belongs to the individuals and communities described in the studies included. Research outputs will help to inform culturally appropriate health interventions aimed at primary and secondary prevention of stroke in Indigenous Peoples.

### Definitions

#### Developed Countries

We identified developed countries as those with a score of ≥0.8 on the United Nations Development Program 2018 Human Development Index (HDI), indicating a “Very High HDI” (Appendix 1) (58). Thus, when we refer to “developed countries” throughout the manuscript, we specifically refer to countries with a Very High HDI. The term “developed countries” is utilized by the United Nations (59), though we acknowledge that this term could be considered somewhat outdated.

The HDI scoring system takes into account overall life expectancy, literacy and gross national income, but does not reflect social inequalities, poverty, security or empowerment (58). Some countries with high HDI scores have minority Indigenous populations, many of whom do not experience the same levels of socioeconomic advantage experienced by the non-Indigenous population of the same region (4, 5, 60). We shall also consider all states of limited recognition with both a high estimated Human Development Index and one or more identifiable Indigenous populations (Appendix 1) (61).

#### Indigenous Populations

We defined Indigenous populations according to the World Health Organisation (WHO) definition, namely “communities that live within, or are attached to, geographically distinct traditional habitats or ancestral territories, and who identify themselves as being part of a distinct cultural group, descended from groups present in the area before modern states were created and current borders defined. They generally maintain cultural and social identities, and social, economic, cultural and political institutions, separate from the mainstream or dominant society or culture” (2). We acknowledge that the Indigenous status of some of the Indigenous populations included in this study may not be officially recognized within their own countries.

No comprehensive list of Indigenous populations of developed countries exist. Consequently, identification (Appendix 1) was based on terms outlined in the UN Permanent Forum on Indigenous Issues (UNPFII) (62) and a range of additional sources. We acknowledge that our search string, by necessity, incorporated certain outdated and potentially discriminatory terms (i.e., Eskimo, Lapp) to avoid omission of any relevant studies. In our search strategy, we also included terms related to geographic regions (i.e., Circumpolar) where a high proportion of Indigenous people reside. In this study, we will refer to specific nations, tribal groups, or peoples. Where this is not possible, we use nation- or region-specific terms, or the generic term, Indigenous Peoples; we respectfully acknowledge that these may not be the preferred terms for use.

#### Stroke

Cases of stroke will be defined using either:

1. The WHO clinical definition of stroke, inclusive of ischemic stroke, spontaneous intracerebral hemorrhage and non-traumatic subarachnoid hemorrhage, and excluding transient ischemic attacks (63); or
2. International Classification of Diseases definition (including the full range or subsets of ICD-10 codes I60-I69, or their equivalent ICD-9 codes) (64).

#### Population-Based study

A study in which the denominator of the study represents the whole population of a given area and case ascertainment, ideally included overlapping sources of information about all hospitalized, non-hospitalized, fatal and non-fatal stroke events.

#### Incident Stroke Events

For the purposes of this study, an incident event is defined as first-ever-in-a-lifetime stroke; to be used when determining incidence rates. The methodology for how this is determined will differ between studies.

#### Total Stroke Events

All strokes occurring within the study period including first-ever and recurrent stroke events; to be used to determine attack rates (the rate of all stroke events within the given population).

#### Standard Population

The proportional age distributions within a population used as weights to create age-standardized statistics (65, 66).

## Materials and Methods

### Eligibility Criteria

#### Inclusion

- Language of publication: All
- Year of publication: Articles/reports published from 1990-current.
- Study designs:
  ° Observational studies of the incidence of stroke, with the following characteristics:
    — Population-based
      · Numerator: hospitalized and non-hospitalized, fatal and non-fatal stroke cases (studies using WHO STEPwise steps 2 and 3 approach for stroke surveillance are allowed) (67).
      · Denominator: the general population in a given catchment or geographical area rather than a non-representative population subset (e.g., a clinically defined patient group).
    —Either prospective or retrospective data collection.
- Data sources used in the studies included:
  ° Population-based active case finding of fatal and non-fatal cases.
  °Administrative data.
  °Hospital-based stroke events covering complete populations (though not necessarily including non-hospitalized stroke deaths, to allow for broader study inclusion).
  °Community-based household stroke surveys.
  °Disease registry, if near-comprehensive coverage is available for stroke.
  °Other sources, deemed appropriate for comprehensive population coverage.
  —Participants:
  °Indigenous and non-Indigenous populations of developed countries.
    —If a study includes data on an Indigenous population without a comparator population, we will include this study in the preliminary data collection in case meta-analysis of Indigenous stroke incidence rates by region is deemed appropriate.
  °Age group: Includes participants aged ≥18 years.
  °Sex: Studies will be included irrespective of whether or not stratification is provided by sex.
  °Geographical: Studies will be included when data are provided at a national, regional, or city/community-specific level, or by levels of urbanization/remoteness.
- Outcome measures:
  °Stroke incidence rates: Ideally, we require availability of crude age-specific (5- or 10-year age bands) incidence rates or numbers of stroke events and population denominators sufficient to calculated age-standardized incidence rates of stroke. However, as this may exclude many studies, we will maintain flexibility to include studies with wider age bands;
  °Stroke attack rates: as above;
  °Rate ratios: Given that methods will vary greatly, we will compare ratios of incidence and/or attack rates (Indigenous vs. non-Indigenous), where available or calculable.
- Stroke types:
  °We will include studies that provide data on total stroke only and/or on stroke type. For comparison incidence of pathological types of stroke, the study subjects should ideally have neuroimaging verification of the type of stroke in ≥70% of subjects (68, 69), although we will also accept administrative data using International Classification of Diseases (ICD) classification.
- Publication type:
  °Original peer-reviewed research published as a letter, abstract, or original scientific report which include sufficient data to meet the above inclusion criteria.

#### Exclusion

- Study design:
  °Study designs that do not provide sufficient information to derive incidence rates. These will include:
    ■ Case-control studies which do not have population-based ascertainment, case report/series, qualitative, review, editorial, intervention, experiment, program evaluation;
    ■ Cohort studies where the participants are not representative of the population at risk of stroke;
    ■ Prevalence studies.
- Study population:
  °Studies including insufficient data to derive incidence rates of stroke in Indigenous populations. These will include:
    ■ Articles where Indigenous data were only reported as baseline characteristics, were combined with other ethnic groups, or where few (<10) Indigenous cases were reported;
    ■ Where there is a clinical/diagnostic subpopulation (i.e., stroke incidence in a select group of patients with a pre-existing condition such as atrial fibrillation) rather than derived from the general population;
    ■ Studies not reporting age-specific stroke events and/or age/sex-specific structure of the denominator sufficient to calculate incidence and mortality rates per year.
- Studies published before 1990;
- Studies not peer-reviewed;
- Rates and count data only presented graphically, with insufficient data to derive incidence rates of stroke in Indigenous populations (after attempts to obtain the data from authors);
- Studies of stroke mortality only;
- Studies of self-report only (survey).

### Information Sources

1. PubMed
2. EMBASE via OVID
3. Global Health via OVID

### Search Strategy

Two authors will independently validate the search strategy, using the search terms outlined in Appendix 2, based on concepts in the three domains of: (1) Population: Indigenous and non-Indigenous adults in developed countries, (2) Outcome: incidence, and (3) Outcome: stroke. The reviewers will perform searches of database-specific controlled vocabulary and synonymous or related text words of the title, abstract and author-selected keywords in each database as applicable. They will hand-search the reference lists of included studies to identify additional papers.

### Study Records

#### Data Management

Records retrieved from the searches will be cataloged in EndNote^®^. Duplicates will be removed by automation, supplemented with manual checking.

#### Selection Process

Using Covidence^®^, titles and abstracts of articles will be screened for relevance by at least two reviewers using a three-question screening process, as described below. Disagreements will be resolved through consensus with a third independent reviewer. Reasons for rejection will be recorded in Covidence^®^. Articles deemed relevant will undergo full-text screening.

The title/abstract screening process includes three yes/maybe/no questions based on the study inclusion criteria. If all questions are answered in any combination of “yes” or “maybe” for a given article, it will be included in the full-text review. If any questions are answered 'no' the article will be excluded. When the questions cannot be answered based on the abstract, the article will be included in the full-text review. The following questions will be used to assess eligibility:

1. Does the article study an identifiable Indigenous population of a developed country?
2. Does the article include an identifiable Indigenous population of a developed country?
3. Does the study have an observational population-based design as defined by the inclusion criteria?
4. Does the article contain incidence (or attack) rates of stroke for the Indigenous population OR contain original count and population data, thereby allowing for calculation of incidence rates?

During the title and abstract screening stage, we will exclude papers based on the follow-criteria:

1. Duplicates;
2. Not peer-reviewed;
3. Study clearly unrelated to stroke/cardiovascular events in human subjects;
4. Study clearly not including population-based data from one of the specified (HDI ≥ 0.8) countries.

During the full-text review stage, we will further exclude papers based on the following related criteria:

1. Did not incorporate details of the incidence or attack rates of stroke (or provide sufficient data to allow the calculation of incidence or attack rates of stroke);
2. Did not include separate details of a specific Indigenous population;
3. Did not include, at minimum, age groups between 35 and 64 years;
4. Did not include an identifiable Indigenous population at the individual or ecological level;
5. Did not include crude, age-specific or adjusted incidence (or attack) rates of stroke or sufficient data (count and population) in the numerator and denominator to estimate rates for an Indigenous population;
6. Provided rates in graphical form only;
7. Did not comprise original research to determine incidence rates (i.e., review articles); and
8. Provided details of incidence (or attack) rates from the same dataset, and with a period of overlap, as another paper that had more comprehensive data (i.e., the article included a shorter period of observation).

### Data Collection Process

Article details will be extracted independently by reviewers from selected papers into a predesigned template in Microsoft Excel (Appendix 3). Variables will include author, year of publication, region, Indigenous population, study design and sample characteristics. Studies will be grouped according to the type(s) of epidemiological data on stroke presented. We anticipate that the content of this data extraction list will be final, but we will pilot this and make changes as necessary.

### Data Items

Article details will be extracted independently by reviewers from selected papers into a predesigned template in Microsoft Excel. Studies will be grouped according to the type(s) of epidemiological data on stroke presented. We anticipate that the content of this data extraction list will be final, but we will pilot it and make changes as necessary.

### Outcomes and Prioritization

#### Primary

Crude, age-specific or age-standardized incidence (first-ever-in-a-lifetime) of stroke in Indigenous and non-Indigenous populations – standardized to WHO population (65, 66).

#### Secondary

Crude, age-specific or age-standardized attack rates (all cases) of stroke; attack rate ratio; case-fatality.

#### Age Standardization

Where age-specific rates are provided, the age structure of the WHO Standard Population will be used to calculate age-standardized stroke incidence (and attack) rates (63). Additionally, age-standardized rates published in the papers using other standard populations will be recorded (if available) recognizing these rates may not be comparable between populations.

### Comparisons

We will examine differences by age, sex and Indigenous status. We will not directly compare the incidence of stroke between different Indigenous populations because methods differ between countries with regard to the recognized definition of Indigenous status, how these data are collected and ascertainment in Indigenous data (13). Furthermore, through consultation with Indigenous researchers and stakeholders, we were advised that certain Indigenous groups do not wish to be compared to other Indigenous populations. Therefore, we will not make any attempt to draw comparisons between different Indigenous groups.

### Risk of Bias in Individual Studies

Risk of bias analysis will be conducted for all research papers. Original research published as an abstract or research letter only will be identified with an asterisk (^*^) in the tables.

The validity of study hypotheses on epidemiological indices of Indigenous stroke will be assessed in applicable cases using the Newcastle-Ottawa Scale (NOS) and reported according to the layout of Table 2 (70). This scale was selected due to its utility in assessing the quality of observational studies.

**Table 2:** Risk of bias assessment in individual studies, using the Newcastle-Ottawa Scale^*^.

**Studies**	**Selection**	**Comparability**	**Outcome/exposure**	**Total score**
Study 1				
Study 2				
Study 3				
Study 4				

^*^Newcastle-Ottawa Scale (70) for non-randomized studies, the maximum score for all fields is 9 (selection 4, comparability 2, outcomes/exposure 3).

In addition to the NOS, we will assess whether each study of stroke incidence was conducted according to “ideal” study criteria, described by Sudlow and Warlow (71) in 1996 (Table 3). Although these were later updated by Feigin and Vander Hoorn in 2004 (72) and then again by Feigin et al. in 2018 (68), we will use the original Sudlow and Warlow criteria (71), given the later updates may have been published after the selected studies had commenced.

**Table 3:** Criteria for Basic and Advanced Population-Based Stroke and TIA Incidence Studies.

	**Study 1**	**Study 2**	**Study 3**	**Study 4**	**Study 5**	**Study 6**
**Standard definitions**						
World Health Organization clinical definition	Yes			Yes		Yes
First-ever-in-a-lifetime stroke						Yes
**Standard methods**						
Complete population-based case ascertainment, based on multiple overlapping sources	Yes			Yes		Yes
Prospective study design, ideally with “hot pursuit” of cases	Yes	Yes		Yes		Yes
Large, well-defined, stable population						Yes
Reliable method for estimating denominator						Yes
**Standard data-presentation**						
Whole years of data	Yes					Yes
Not >5 years of data averaged together						Yes
Men and women presented separately			Yes			Yes
Include ages up to ≥85 years if possible						Yes
Standard mid-decade age bands (e.g., 55 to 64 years) used in publications						Yes
Unpublished 5-year age bands available for comparison with other studies						Yes
Presentation of 95% confidence intervals around incidence rates						Yes
**“Ideal” study (i.e., meets all criteria)**	**No**	**No**	**No**	**No**	**No**	**Yes**

*Adapted from Sudlow and Warlow (71)*.

#### Quality of Reporting in Individual Studies

Studies will be assessed using the consolidated criteria for strengthening reporting of health research involving Indigenous Peoples (CONSIDER) checklist, a collaborative synthesis and prioritization of existing national and international statements and guidelines (Table 4) (22). We will examine whether individual studies specifically addressed these eight domains. As we recognize the limitations of determining this solely based on published material, if at least one of the items within a given domain is addressed, we will consider that the study has met criteria for that domain. We will report a proportion of studies that address these individual domains, using a radar plot. Total number of studies permitting, we will generate similar plots for different time epochs and different regions.

**Table 4:** Consider checklist: items to include when reporting health research involving Indigenous Peoples.

**Governance**	
1.	Describe partnership agreements between the research institution and Indigenous-governing organization for the research.
2.	Describe accountability and review mechanisms within the partnership agreement that addresses harm minimization.
3.	Specify how the research partnership agreement includes protection of Indigenous intellectual property and knowledge arising from the research.
**Prioritization**	
4.	Explain how the research aims emerged from priorities identified by either Indigenous stakeholders, governing bodies, funders, non-government organizations, stakeholders, consumers, and empirical evidence.
**Relationships (Indigenous stakeholders/participants and Research team)**	
5.	Specify measures that adhere and honor Indigenous ethical guidelines, processes, and approvals for all relevant Indigenous stakeholders.
6.	Report how Indigenous stakeholders were involved in the research processes.
7.	Describe the expertise of the research team in Indigenous health and research.
**Methodologies**	
8.	Describe the methodological approach of the research including a rationale of methods used and implication for Indigenous stakeholders.
9.	Describe how the research methodology incorporated consideration of the physical, social, economic and cultural environment of the participants and prospective participants.
**Participation**	
10.	Specify how individual and collective consent was sought to conduct future analysis on collected samples and data.
11.	Described how the resource demands (current and future) placed on Indigenous participants and communities involved in the research were identified and agreed upon.
12.	Specify how biological tissue and other samples including data were stored, explaining the processes of removal from traditional lands, if done, and of disposal *(Not applicable for this current study)*.
**Capacity**	
13.	Explain how the research supported the development and maintenance of Indigenous research capacity.
14.	Discuss how the research team undertook professional development opportunities to develop the capacity to partner with Indigenous stakeholders.
**Analysis and interpretation**	
15.	Specify how the research analysis and reporting supported critical inquiry and a strength-based approach inclusive of Indigenous values.
**Dissemination**	
16.	Describe the dissemination of the research findings to relevant Indigenous governing bodies and peoples.
17.	Discuss the process for knowledge translation and implementation to support Indigenous advancement.

*Adapted from Huria et al. (22) with permissions*.

### Data Synthesis

We will conduct a narrative review of studies that provide estimates of incidence (and attack) rates and rate ratios. Results will be synthesized in figures and tables. These will include descriptions of data sources, populations, methodology, and findings. We anticipate heterogeneity in the data from included studies (population data sources used, study period, age and sex stratification, population size, and comparators). Therefore, we are not planning to conduct a meta-analysis across countries. We will attempt this within countries, subject to (1) a sufficient number of studies and (2) comparable design of studies.

1. Age standardized rate
  - Use data provided
  - Recalculate according to WHO population
2. Age standardized rate ratio
  - Use data provided
  - WHO standard rate ratio (if age-specific numbers and denominator available)
3. Trends over time (if available)
4. A map showing where these studies arise and where data are unavailable.

### Meta-Analysis and Meta-Bias(ES)

Due to anticipated heterogeneity of data, and as we are not making comparisons between different Indigenous populations, we expect little or no potential for meta-analysis or assessment of meta-biases across studies. However, we will aim to determine whether there is any role for meta-analysis in this setting. Specifically, if there are a sufficient number of “gold-standard” incidence studies with comparable methodology for the same Indigenous population to allow for comparison, meta-analysis (stratified by jurisdiction) may be undertaken.

### Confidence in Cumulative Evidence

Due to anticipated heterogeneity of data, and as we are not making comparisons between different Indigenous populations, we will not be reporting confidence in cumulative evidence.

## Discussion

In this study, we will undertake the first systematic review assessing disparities in stroke incidence in Indigenous populations of developed countries worldwide. Our findings will be placed in the broader context of health research involving Indigenous Peoples, recognizing (though not aiming to measure) the social determinants of health. We anticipate that the incidence of stroke will generally, though not uniformly, be greater in Indigenous populations than their respective non-Indigenous populations. Our systematic methodology will offer critical insights into gaps in the availability and quality of data on stroke incidence in Indigenous populations, as well as methodological challenges and suitable approaches to obtaining valid estimates. High-quality data are imperative for informing effective health interventions, and appropriate consultation and insight from Indigenous stakeholders critical to their efficacy.

Indigenous health research is often framed placing those of white majorities (for example, non-Hispanic ethnicity in the USA) as the referent category, with the implication of this being the “normal” group. This largely arises because the referent group is ideally a larger group than the comparison group, thereby providing estimates that are more robust. An alternative view is that Indigenous or minority groups could be referent, and other populations could be the comparison group. Use of this strategy has to be considered in the context of the population size.

### Strengths and Limitations

A major strength of the planned review is the input and oversight from the outset of Indigenous researchers from around the world into the conceptualization and study design. These Indigenous researchers represent a range of Indigenous cultures, thereby providing a broad perspective of Indigenous cultures. This new international partnership will strengthen collaboration, with potential to develop innovative ways of working in the global Indigenous stroke space. Not only will we provide information on the incidence rates of stroke in various Indigenous populations of developed countries worldwide, but we will also appraise whether the studies in which these data are published appear to have been undertaken with the appropriate cultural lens. Consequently, the review will not only provide insight into disparities that need to be addressed but will also contribute to establishing frameworks that optimize the benefit of future research for Indigenous Peoples.

There are also several limitations in this study. In our protocol, we have endeavored to incorporate search terms that identify as many as possible of the various Indigenous Peoples of developed countries; however, we acknowledge that our list cannot realistically be exhaustive. We have based our definition of developed countries on the HDI, including only those countries fulfilling criteria for “very high human development,” in which excellent health indices and life-expectancy of the general population typically do not extend to Indigenous minority groups. However, we acknowledge that the chosen cut-point for HDI dichotomization is ultimately arbitrary, and that the term “developed countries” could be considered somewhat outdated and colonialistic, despite being the current preferred term used by the United Nations. Additionally, we anticipate substantial heterogeneity in the number of studies per region, data quality, and methodologies (including case ascertainment and identification of Indigenous status) of included studies. We are also not examining the “gray literature”; therefore, the data retrieved will be less comprehensive than if we included this literature. However, it is likely that exclusion of the gray literature will result in greater overall accuracy, given the rigorous methods inherent in peer review processes. Assessment criteria for stroke incidence studies (71) may not be appropriate for those studies conducted prior to the publication of these recommendations, so will be acknowledged when this occurs. Quality assessment using the NOS may be limited by bias due to inter-observer variation (73). Ascertaining the extent of Indigenous engagement based on information provided explicitly within the published studies will be limited by numerous factors, such as word count limits, authorship criteria and other similar publication constraints (74). Additionally, many of these studies will have been published prior to the development of the CONSIDER statement (or similar guidelines), so we may observe a change in reporting over time. Indeed, deficits in this area may highlight a necessity for changes in editorial policies to help promote greater methodological transparency. Finally, while development of this protocol has benefitted from substantial input and oversight provided by researchers across many countries, the study was originally conceptualized by Australian researchers, who are most familiar with Indigenous health issues and research challenges in the Australian context. Thus, we acknowledge that aspects of our commentary are inevitably grounded in this perspective and may not necessarily be applicable to other Indigenous contexts worldwide. As the study progresses, we anticipate that our perspective will be broadened, particularly given the broader oversight from the Indigenous Advisory Board.

### Dissemination Plan

We will submit the completed review for publication in a peer-reviewed journal. Prior to submission, the manuscript will be scrutinized by the Indigenous Advisory Board, who will assess and advise whether all aspects of the study have been conducted with the priorities of the Indigenous Peoples remaining central throughout the process. Both this protocol and the completed review manuscript will also be included in the doctoral thesis of one of the authors (AHB). We will present the findings at professional fora and Indigenous health conferences. Further, we will communicate results in the form of oral presentations to relevant stakeholder and consumer groups, including Indigenous communities and non-government organizations (i.e., National Stroke Foundations.). We will also prepare accessible summary reports using visual graphics and interactive maps and will use these in our presentations and printed materials. This process will be undertaken with guidance and oversight from our Indigenous Advisory Board, and additional knowledge sharing mechanisms will be discussed during the progress of the systematic review.

## Ethics Statement

Ethical review and approval was not required for the study on human participants in accordance with the local legislation and institutional requirements. Written informed consent for participation was not required for this study in accordance with the national legislation and the institutional requirements.

## Author Contributions

AHB, AD, JK, TK, and AT devised the project and main conceptual ideas with the assistance of all co-authors. AHB, AD, and JW developed the search strategy and took the lead in preparing the manuscript, with substantial input from JK, AT, TK, and AS-D. All authors (including AS-D, SS, BB-A, RK, VF, DB, AR, CM, CZ, LC, LB, DZ, WL, DT, SA, MP, AB, DW, and MH) provided critical input that helped shape and develop the protocol and manuscript. AHB and LC determined the statistical methods. The Indigenous Advisory Board (AD, CM, MH, LB, AB, and DW) provided oversight into all aspects of the protocol development. JK, TK, and AT supervised the project. All authors contributed to the article and approved the submitted version.

## Conflict of Interest

The authors declare that the research was conducted in the absence of any commercial or financial relationships that could be construed as a potential conflict of interest. The Handling Editor declared a past co-authorship with two of the authors, AD and MP.
