# Supplementary material for: The Incidence of Stroke in Indigenous Populations of Countries With a Very High Human Development Index: A Systematic Review Protocol
Source: Front Neurol. 2021 Apr 22;12:661570. doi: 10.3389/fneur.2021.661570 (PMC8100239; doi:10.3389/fneur.2021.661570)
Supplement: Supplementary file 1 [file Data_Sheet_1.docx]

Supplementary Material

# Appendix 1

Countries identified as receiving a Human Development Index (HDI) ≥0.8 in 2018, with relevant Indigenous populations. This level is defined by the United Nations Development Programme's as having "very high human development".

| **HDI** | **Country*** | **FINAL TERMS CHOSEN** |
| --- | --- | --- |
| 1 | Norway | Sami/Sámi/Saami  Lapp/Lappish/Laplander |
| 2 | Switzerland | - |
| 3 | Australia | Aboriginal  Torres Strait Islander |
| 4 | Ireland | - |
| 5 | Germany | - |
| 6 | Iceland | - |
| 7 | Hong Kong, China (SAR) | - |
| 7 | Sweden | Sami/Sámi/Saami  Lapp/Lappish/Laplander |
| 9 | Singapore | Malay Singaporeans/Singaporean Malays |
| 10 | Netherlands | Frisian |
| 11 | Denmark | Inuit  Kalaallit |
| 12 | Canada | Aboriginal  First Nations  Indians  Inuit/Eskimo/Nunavik/Nunavimmiut/Nunavut  Métis |
| 13 | United States | Alaska Athabaskan  Alaska Native  Aleut  Alutiiq  Cup'ik  Eyak  Inuit/Eskimo  Native Americans/American Indians  Native Hawai'ians  Navajo  Tlingit-Haida  Tsimshian Natives  Yup'ik/Yupik |
| 14 | United Kingdom | - |
| 15 | Finland | Sami/Sámi/Saami |
| 16 | New Zealand | Māori/Maori |
| 17 | Belgium | - |
| 17 | Liechtenstein | - |
| 19 | Japan | Ryukyuans/Okinawans  Ainu |
| 20 | Austria | - |
| 21 | Luxembourg | - |
| 22 | Israel | Bedouin |
| 22 | Korea (Republic of) | - |
| 24 | France | - |
| 25 | Slovenia | - |
| 26 | Spain | Basques |
| 27 | Czechia | - |
| 28 | Italy | - |
| 29 | Malta | - |
| 30 | Estonia | Seto |
| 31 | Greece | - |
| 32 | Cyprus | - |
| 33 | Poland | - |
| 34 | United Arab Emirates | - |
| 35 | Andorra | - |
| 35 | Lithuania | - |
| 37 | Qatar | - |
| 38 | Slovakia | - |
| 39 | Brunei Darussalam | Dusun  Murut  Kedayan  Iban  Tutong  Penan |
| 39 | Saudi Arabia | - |
| 41 | Latvia | Livs |
| 41 | Portugal | - |
| 43 | Bahrain | - |
| 44 | Chile | Atacameño/Atacama  Aymara  Colla  Diaguita  Kawésqar/Alacalufe  Lickanantay  Mapuche  Quechua  Rapanui  Yamana/Yámana/Yagán/Yagan |
| 45 | Hungary | - |
| 46 | Croatia | - |
| 47 | Argentina | Atacama  Avá-Guaraní  Aymara  Chané  Charrúa  Chiriguano  Choroti/Chorote  Chulupí  Colla/Kolla  Comechingon  Diaguita-Calchaquí  Guaraní/Guaranies/Mbyá  Huarpe  Iyojwaja (Chorote)  Lule  Mapuche  Mapudungun  Mataco  Mocovi/Mocoví  Nivacklé (Chulupí)  Ocloya  Omaguaca  Ona  Qom (Toba)  Quechua  Rankulche  Selk'namgon  Tapy'y (Tapiete)  Tehuelche  Toba  Tonocoté  Tupí-Guaraní  Vilela  Wichi/Wichí/Mataco  Yamana |
| 48 | Oman | - |
| 49 | Russian Federation | Ainu/Aynu,  Aluet, Khanty  Chelkans  Chukchi  Chukotka  Chulyms,  Chuvan  Dolgan,  Enets, Yupiq (Eskimo)  Evenk/Evenki  Even (Lamuts)  Itelmen  Izvatas (the Izhma Komi)  Kamchadal/Kamachatka  Kerek  Kets  Khanty  Koryak  Kumandins  Laks/Laki  Mansi  Nagaibaks  Nanai  Negidals  Nenets/Samoyed  Komi  Nganasan  Nivkh  Oroch  Orok  Sami/Saami  Selkup  Shor  Soyots  Taz  Telengits  Teleuts  Tofalars (Tofa)  Tozhu  Tubalars  Udege  Uilta (Oroks)  Ulchi  Vainakh  Veps  Volga Finns  Yukagir  Yupiq |
| 50 | Montenegro | - |
| 51 | Bulgaria | - |
| 52 | Romania | - |
| 53 | Belarus | - |
| 54 | Bahamas | Taino/Lucayan |
| 55 | Uruguay | Charrúa  Guaraní Mbyá |
| 56 | Kuwait | - |
| 57 | Malaysia | Anak Negeri  Natives  Orang Asli/Aslian  Orang Ulu/Dayak |
| 58 | Barbados | Taino/Arawak  Kalinago |
| 58 | Kazakhstan | - |
| N/A | Taiwan | Ami  Atayal  Paiwan  Bunun  Puyuma  Rukai  Tsou  Saisiyat  Tao (Yami)  Thao  Kavalan  Taroko/Truku  Sakizaya |
|  | **GENERAL TERMS** | Aboriginal  Amerind  First Peoples  Indigenous Peoples  Native Peoples  Tribal Peoples |
|  | **Notes on Terms** | *Includes states of limited recognition with a both a high estimated Human Development Index and one or more identifiable Indigenous populations  We acknowledge that our search string incorporated certain outdated and potentially discriminatory terms (i.e. Eskimo, Lapp) used to describe these Peoples; these terms were included to avoid omission of any relevant studies. |

# Appendix 2: Search Strategy

| Concept Domain | PubMed | OVID-EMBASE | OVID-Global Health |
| --- | --- | --- | --- |
| Population: Indigenous and non-Indigenous adults in developed nations | **Controlled Subject Headings**  "American Native Continental Ancestry Group" [MeSH Terms] OR  "Oceanic Ancestry Group" [MeSH Terms] OR  "Indigenous Peoples" [MeSH Terms] OR  "Health Services, Indigenous" [MeSH Terms] OR  "arctic regions" [MeSH Terms] OR  "ethnic groups" [MeSH Terms] OR  **Synonyms (Title, Abstract, Keywords)**  Aborigin*[TIAB] OR  Ainu[TIAB] OR  Alacalufe[TIAB] OR  "Alaska Athabaskan" [TIAB] OR  Aluet[TIAB] OR  "Alaska Native" [TIAB] OR  Alutiiq[TIAB] OR  "American Indian" [TIAB] OR  Amerind[TIAB] OR  Amis[TIAB] OR  "Anak Negeri" [TIAB] OR  Arawak[TIAB] OR  Atacama[TIAB] OR  Atacameño[TIAB] OR  Atayal[TIAB] OR  "Avá-Guaraní"[TIAB] OR  Aymara[TIAB] OR  Aynu[TIAB] OR  Basque*[TIAB] OR  Bedouin[TIAB] OR  Bunun[TIAB] OR  Chané[TIAB] OR  Charrua[TIAB] OR  Chelkan*[TIAB] OR  Chiriguano[TIAB] OR  Chorote[TIAB] OR  Choroti[TIAB] OR  Chukchi[TIAB] OR  Chukotka*[TIAB] OR  Chulupí[TIAB] OR  Chulyms[TIAB] OR  Chuvan*[TIAB] OR  Circumpolar[TIAB] OR  Colla[TIAB] OR  Comechingón[TIAB] OR  Cupik[TIAB] OR  Cup'ik[TIAB] OR  Diaguita[TIAB] OR  Dolgan*[TIAB] OR  Dusun[TIAB] OR  Ethnic[TIAB] OR  Enets[TIAB] OR  Eskimo*[TIAB] OR  Evenk*[TIAB] OR  "Evens"[TIAB] OR  Eyak[TIAB] OR  "First nation" [TIAB] OR  "First nations" [TIAB] OR  "First Peoples" [TIAB] OR  Frisian[TIAB] OR  Guaraní[TIAB] OR  Huarpe*[TIAB] OR  Iban[TIAB] OR  Indians[TIAB] OR  Indigenous[TIAB] OR  "Indigenous Peoples"[TIAB] OR  Inupia*[TIAB] OR  Inuit[TIAB] OR  Inuvialuit[TIAB] OR  Itelmen*[TIAB] OR  Iyojwaja[TIAB] OR  Izvatas[TIAB] OR  Kalaallit[TIAB] OR  Kalingo*[TIAB] OR  Kamchadal*[TIAB] OR  Kavalan[TIAB] OR  Kawesqar[TIAB] OR  Kedayan[TIAB] OR  Kerek*[TIAB] OR  Kets[TIAB] OR  Khanty[TIAB] OR  Kolla[TIAB] OR  Komi[TIAB] OR  Koryak*[TIAB] OR  Kumandins[TIAB] OR  Laki[TIAB] OR  Laks[TIAB] OR  Lapp[TIAB] OR  Lappish[TIAB] OR  Laplander[TIAB] OR  Lickanantay[TIAB] OR  Livs[TIAB] OR  Lule[TIAB] OR  Lukayan[TIAB] OR  "Malay Singaporean"[TIAB] OR  "Malay Singaporeans" [TIAB] OR  Mansi[TIAB] OR  Maori[TIAB] OR  Mapuche[TIAB] OR  Mapudungun[TIAB] OR  Mataco[TIAB] OR  Mbyá[TIAB] OR  Metis[TIAB] OR  Mocovi[TIAB] OR  Murut[TIAB] OR  Nagaibaks[TIAB] OR  Nanai[TIAB] OR  "Native American" [TIAB] OR  "Native Americans" [TIAB] OR  "Native Hawaiian" [TIAB] OR  "Native Hawai'ian" [TIAB] OR  "Native Hawai'ians" [TIAB] OR  "Native Hawaiians" [TIAB] OR  "Native people" [TIAB] OR  "Native peoples" [TIAB] OR  "Native population" [TIAB] OR  "Native populations" [TIAB] OR  Natives[TIAB] OR  Navaho[TIAB] OR  Navajo[TIAB] OR  Negidals[TIAB] OR  Nenets[TIAB] OR  Nganasan*[TIAB] OR  Nivacklé[TIAB] OR  Nivkh*[TIAB] OR  Nunavik[TIAB] OR  Nunavimmiut[TIAB] OR  Nunavut[TIAB] OR  Ocloya[TIAB] OR  Okinawan*[TIAB] OR  Omaguaca[TIAB] OR  Ona[TIAB] OR  "Orang Asli" [TIAB] OR  "Orang Aslian" [TIAB] OR  "Orang Ulu" [TIAB] OR  Oroch[TIAB] OR  Orok[TIAB] OR  Paiwan[TIAB] OR  Penan[TIAB] OR  Puyuma[TIAB] OR  Qawasqar[TIAB] OR  Qom[TIAB] OR  Quechua[TIAB] OR  Qulla[TIAB] OR  Rankulche[TIAB] OR  Rapanui[TIAB] OR  Rukai[TIAB] OR  Ryukyuan*[TIAB] OR  Saisiyat[TIAB] OR  Sakizaya[TIAB] OR  Sakha[TIAB] OR  Saami[TIAB] OR  Sami[TIAB] OR  Samoyed[TIAB] OR  Selknamgon[TIAB] OR  Selk'namgon[TIAB] OR  Selkup[TIAB] OR  Seto[TIAB] OR  Shor[TIAB] OR  Soyot*[TIAB] OR  Siberia[TIAB] OR  "Singaporean Malay" [TIAB] OR  "Singaporean Malays" [TIAB] OR  Soyots[TIAB] OR  Skolt[TIAB] OR  Taino[TIAB] OR  Tao[TIAB] OR  Tapiete[TIAB] OR  Tapyy[TIAB] OR  Tapy'y[TIAB] OR  Taroko[TIAB] OR  Taz[TIAB] OR  Tehuelche[TIAB] OR  Telengits[TIAB] OR  Teleuts[TIAB] OR  Thao[TIAB] OR  Tlingit[TIAB] OR  Tofalars[TIAB] OR  Tofa[TIAB] OR  Toba[TIAB] OR  "Tonocoté"[TIAB] OR  "Torres Strait Islander" [TIAB] OR  "Torres Strait Islanders" [TIAB] OR  Tozhu[TIAB] OR  Tribal[TIAB] OR  Tribe*[TIAB] OR  Truku[TIAB] OR  Tsimshian[TIAB] OR  Tsou[TIAB] OR  Tubalars[TIAB] OR  Tutong[TIAB] OR  "Tupí-Guaraní"[TIAB] OR  Udege[TIAB] OR  Uilta[TIAB] OR  Ulchi[TIAB] OR  Vainakh[TIAB] OR  Veps[TIAB] OR  Vilela[TIAB] OR  "Volga Finns" [TIAB] OR  Wichi[TIAB] OR  "Yagán"[TIAB] OR  Yamana[TIAB] OR  Yami[TIAB] OR  Yukagir*[TIAB] OR  Yupik[TIAB] OR  Yup'ik[TIAB] OR  Zuni[TIAB] | **Controlled Subject Headings**  exp Indigenous people/ OR  exp Asian continental ancestry group/ OR  exp Oceanic ancestry group/ OR  exp ethnic group/ OR  **Synonyms (Title, Abstract, Keywords)**  (Aborigin* or Ainu or "Alaska Athabaskan" or Alacalufe or Aluet or "Alaska Native" or Alutiiq or "American Indian" or Amerind or Amis or "Anak Negeri" or Arawak or Atacama or Atacameno or "Atacameño" or Atayal or "Avá-Guaraní" or Ava-Guarani or Aymara or Aynu or Basque* or Bedouin or Bunun or Chane or "Chané" or Charrua or Chelkan* or Chiriguano or Chorote or Choroti or Chukchi or Chukotka or Chulupi or "Chulupí" or Chulyms or Chuvan* or Circumpolar or Colla or Comechingon or "Comechingón" or "Cup'ik" or Diaguita or Dolgan* or Dusun or Ethnic or Enets or Eskimo* or Evenk* or "Evens" or Eyak or "First nation" or "First nations" or "First Peoples" or Frisian or Guarani or "Guaraní" or Huarpe* or Iban or Indians or Indigenous or "Indigenous Peoples" or Inupia* or Inuit or Inuvialuit or Itelmen* or Iyojwaja or Izvatas or Kalaallit or Kalingo* or Kamchadal* or Kavalan or Kawesqar or Kedayan or Kerek* or Kets or Khanty or Kolla or Komi or Koryak* or Kumandins or Laki or Laks or Lapp or Lappish or Laplander or Lickanantay or Livs or Lule or Lukayan or "Malay Singaporean" or "Malay Singaporeans" or Mansi or Maori or "Māori" or Mapuche or Mapudungun or Mataco or Mbya or "Mbyá" or Metis or Mocovi or Murut or Nagaibaks or Nanai or "Native American" or "Native Americans" or "Native Hawai'ian" or "Native Hawai'ians" or "Native people" or "Native peoples" or "Native population" or "Native populations" or Natives or Navaho or Navajo or Negidals or Nenets or Nganasan* or Nivackle or "Nivacklé" or Nivkh* or Nunavik or Nunavimmiut or Nunavut or Ocloya or Okinawan* or Omaguaca or Ona or "Orang Asli" or "Orang Aslian" or "Orang Ulu" or Oroch or Orok or Paiwan or Penan or Puyuma or Qawasqar or Qom or Quechua or Qulla or Rankulche or Rapanui or Rukai or Ryukyuan* or Saisiyat or Sakizaya or Sakha or Saami or Sami or "Sámi" or Samoyed or "Selk'namgon" or Selkup or Seto or Shor or Soyot* or Siberia or "Singaporean Malay" or "Singaporean Malays" or Soyots or Skolt or Taino or Tao or Tapiete or "Tapy'y" or Taroko or Taz or Tehuelche or Telengits or Teleuts or Thao or Tlingit or Tofalars or Tofa or Toba or Tonocote or "Tonocoté" or "Torres Strait Islander" or "Torres Strait Islanders" or Tozhu or Tribal or Tribe* or Truku or Tsimshian or Tsou or Tubalars or Tutong or Tupi-Guarani or "Tupí-Guaraní" or Udege or Uilta or Ulchi or Vainakh or Veps or Vilela or "Volga Finns" or Wichi or Yagan or "Yagán" or Yamana or "Yámana" or Yami or Yukagir* or Yupik or "Yup'ik" or Zuni).tw,kw. | **Controlled Subject Headings**  Indigenous people/ or aborigines/ or alaska natives/ or american indians/ or inuit/ or  **Synonyms**  (Aborigin* or Ainu or "Alaska Athabaskan" or Alacalufe or Aluet or "Alaska Native" or Alutiiq or "American Indian" or Amerind or Amis or "Anak Negeri" or Arawak or Atacama or Atacameno or "Atacameño" or Atayal or "Avá-Guaraní" or Ava-Guarani or Aymara or Aynu or Basque* or Bedouin or Bunun or Chane or "Chané" or Charrua or Chelkan* or Chiriguano or Chorote or Choroti or Chukchi or Chukotka or Chulupi or "Chulupí" or Chulyms or Chuvan* or Circumpolar or Colla or Comechingon or "Comechingón" or "Cup'ik" or Diaguita or Dolgan* or Dusun or Ethnic or Enets or Eskimo* or Evenk* or "Evens" or Eyak or "First nation" or "First nations" or "First Peoples" or Frisian or Guarani or "Guaraní" or Huarpe* or Iban or Indians or Indigenous or "Indigenous Peoples" or Inupia* or Inuit or Inuvialuit or Itelmen* or Iyojwaja or Izvatas or Kalaallit or Kalingo* or Kamchadal* or Kavalan or Kawesqar or Kedayan or Kerek* or Kets or Khanty or Kolla or Komi or Koryak* or Kumandins or Laki or Laks or Lapp or Lappish or Laplander or Lickanantay or Livs or Lule or Lukayan or "Malay Singaporean" or "Malay Singaporeans" or Mansi or Maori or "Māori" or Mapuche or Mapudungun or Mataco or Mbya or "Mbyá" or Metis or Mocovi or Murut or Nagaibaks or Nanai or "Native American" or "Native Americans" or "Native Hawai'ian" or "Native Hawai'ians" or "Native people" or "Native peoples" or "Native population" or "Native populations" or Natives or Navaho or Navajo or Negidals or Nenets or Nganasan* or Nivackle or "Nivacklé" or Nivkh* or Nunavik or Nunavimmiut or Nunavut or Ocloya or Okinawan* or Omaguaca or Ona or "Orang Asli" or "Orang Aslian" or "Orang Ulu" or Oroch or Orok or Paiwan or Penan or Puyuma or Qawasqar or Qom or Quechua or Qulla or Rankulche or Rapanui or Rukai or Ryukyuan* or Saisiyat or Sakizaya or Sakha or Saami or Sami or "Sámi" or Samoyed or "Selk'namgon" or Selkup or Seto or Shor or Soyot* or Siberia or "Singaporean Malay" or "Singaporean Malays" or Soyots or Skolt or Taino or Tao or Tapiete or "Tapy'y" or Taroko or Taz or Tehuelche or Telengits or Teleuts or Thao or Tlingit or Tofalars or Tofa or Toba or Tonocote or "Tonocoté" or "Torres Strait Islander" or "Torres Strait Islanders" or Tozhu or Tribal or Tribe* or Truku or Tsimshian or Tsou or Tubalars or Tutong or Tupi-Guarani or "Tupí-Guaraní" or Udege or Uilta or Ulchi or Vainakh or Veps or Vilela or "Volga Finns" or Wichi or Yagan or "Yagán" or Yamana or "Yámana" or Yami or Yukagir* or Yupik or "Yup'ik" or Zuni).mp |
| Outcome: Incidence | **Controlled Subject Headings**  "Epidemiology" [MeSH Subheading] OR  "epidemiologic studies" [MeSH Terms] OR  "Epidemiologic Methods"[MeSH Terms] OR  "Epidemiologic Methods"[MeSH Subheading] OR  "Incidence" [MeSH Terms] OR  "Population" [MeSH Terms] OR  "Population Groups" [MeSH Terms] OR  "registries"[MeSH Terms] OR  "observational study"[Publication Type] OR  **Synonyms (Title, Abstract, Keywords)**  Incidence[TIAB] OR  Epidemiol*[TIAB] OR  Population-based[TIAB] OR  Population[TIAB] OR  Observational[TIAB] OR  Descriptive[TIAB] OR  Register*[TIAB] OR  Registry[TIAB] OR  Registries[TIAB] | **Controlled Subject Headings**  exp epidemiology/ OR  exp epidemiological data/ OR  exp incidence/ OR  exp population/ OR  exp population group/ OR  exp population research/ OR  exp register/ OR  exp observational study/  **Synonyms (Title, Abstract, Keywords)**  (incidence or epidemiol* or population-based or population or observational or descriptive or register* or registry or registries).tw,kw. | **Controlled Subject Headings**  epidemiology/ or disease distribution/ or disease prevalence/ or epidemiological surveys/ or morbidity/ or disease surveys/ or  **Synonyms**  (epidemiol* or incidence or "population-based" or (observational adj2 study) or (observational adj2 studies) or (descriptive adj2 study) or (descriptive adj2 studies) or (longitudinal adj2 study) or (longitudinal adj2 studies) or cohort* or register* or registry or registries).mp. |
| Outcome: Stroke | **Controlled Subject Headings**  "Stroke" [MeSH Terms] OR  "cerebral infarction"[MeSH Terms] OR  "brain ischemia"[MeSH Terms] OR  "brain infarction"[MeSH Terms] OR  "cerebral hemorrhage"[MeSH Terms] OR  "intracranial hemorrhages"[MeSH Terms] OR  "subarachnoid hemorrhage" [MeSH Terms] OR  **Synonyms (Title, Abstract, Keywords)**  Stroke[TIAB] OR  cerebrovasc*[TIAB] OR  CVA[TIAB] OR  "ischemic stroke" [TIAB] OR  "ischaemic stroke" [TIAB] OR  "cerebral infarction"[TIAB] OR  "cerebral ischemia" [TIAB] OR  "cerebral ischaemia" [TIAB] OR  "brain infarction" [TIAB] OR  "brain ischemia" [TIAB] OR  "brain ischaemia" [TIAB] OR  "intracerebral hemorrhage" [TIAB] OR  "intracerebral haemorrhage" [TIAB] OR  "intracranial hemorrhage" [TIAB] OR  "intracranial haemorrhage" [TIAB] OR  ICH[TIAB] OR  "hemorrhagic stroke" [TIAB] OR  "haemorrhagic stroke" [TIAB] OR  "cerebral hemorrhage" [TIAB] OR  "cerebral haemorrhage" [TIAB] OR  "subarachnoid haemorrhage" [TIAB] OR  "subarachnoid haemorrhage" [TIAB] OR  SAH[TIAB] | **Controlled Subject Headings**  exp cerebrovascular accident/ OR  exp cerebrovascular disease/ OR  **Synonyms (Title, Abstract, Keywords)**  (Stroke or "cerebrovasc*" or "cerebral vascular" or CVA or "isch?emic stroke" or "cerebral infarction" or "cerebral isch?emia" or "brain infarction" or "brain isch?emia" or "intracerebral h?emorrhage" or "intracranial h?emorrhage" or ICH or "h?emorrhagic stroke" or "cerebral h?emorrhage" or "subarachnoid h?emorrhage" or SAH).tw,kw. | **Controlled Subject Headings**  exp stroke/ OR  **Synonyms**  (stroke or "cerebrovasc*" or "cerebral vascular" or CVA or "isch?emic stroke" or "cerebral infarction" or "cerebral isch?emia" or "brain infarction" or "brain isch?emia" or "intracerebral h?emorrhage" or "intracranial h?emorrhage" or ICH or "h?emorrhagic stroke" or "cerebral h?emorrhage" or "subarachnoid h?emorrhage" or SAH).mp |
| Limits | From 1990- | From 1990- | From 1990- |
| Notes on syntax | [TIAB] = includes title, abstract, keywords | .tw,kw. = includes title, abstract, keywords | .mp = includes title, abstract, broad terms, heading words (used as no option for "keywords" available) |
| Notes on terms chosen | We acknowledge that our search string incorporated certain outdated and potentially discriminatory terms (i.e. Eskimo, Lapp) used to describe these Peoples; these terms were included to avoid omission of any relevant studies. | | |

**Search strategy:** 1 AND 2 AND 3 applying relevant limits.

# Appendix 3: Data Extraction (Excel Template)

| Publication details |  |
| --- | --- |
| First author |  |
| Year of publication |  |
| Title |  |
| Type | Peer-reviewed paper  Report  Letter  Abstract/poster  Other |
| Focus of study | Stroke – all  IS  ICH  SAH  Hemorrhagic stroke – ICH + SAH combined  Other subclassification of stroke  General cardiovascular disease  Other (specify in comments) |
| Study Setting |  |
| Geographic Setting (country) |  |
| Geographic setting (country/city/region) |  |
| Geographic setting (urban/rural/both) | Urban  Rural  Urban and rural  Other (specify in comments) |
| Geographic coverage (national, subnational, facility, etc.) | Multi-national  National  Subnational, multiple settings: state/city/province/district  Subnational, single setting: state/city/province/district  Other (specify in comments) |
| WHO Region |  |
| 2018 HDI |  |
| Study Population |  |
| Indigenous Peoples, Nation or Tribe(s) |  |
| Source of Indigenous denominator | **SPECIFY** |
| Data sources of Indigenous identification | Self-reported  Indigenous register  Physician identification/Hospital data  Geographic proxy  Language proxy  Other |
| Comparison population |  |
| Source of non-Indigenous denominator | **SPECIFY** |
| Specific population subgroups studied | Total population  Hospitalized patients  Particular ethnic or socioeconomic group (specify in comments)  Other (specify) |
| Reliable method for estimating denominator (census data not more than 5 years old) | Yes  No  Unclear |
| Population/distribution used as the standard (i.e. WHO) | WHO Standard  Other |
| Standard Definition |  |
| Stroke classification | WHO  Oxford/Bamford classification  Other (specify in comments) |
| % who had neuroimaging | x% Indigenous, y% non-Indigenous, z% total  Unclear/undocumented |
| At least 80% verification by computed tomography or magnetic resonance imaging | Yes  No  Unclear |
| Classification of ischemic stroke into subtypes | Yes  No  N/A |
| First-ever-in-a-lifetime and recurrent stroke (separately and combined) | Yes  No |
| How was incident stroke determined? | Administrative data Lookback period (specify lookback)  Clinical history  Other (specify in comments) |
| Study Methodology |  |
| Study period (years incidence figures given) |  |
| Study design | Prospective population-based study  Retrospective population-based study  Other (specify in comments) |
| Methods used for case ascertainment (multiple can be selected) | Medical records – chart review  Medical records – radiological findings  Pre-existing research databases  Administrative data – hospital records  Administrative data – mortality data  Administrative data – emergency data  Administrative data – outpatient data  Primary care sources  Stroke Registry data  Other (specify in comments) |
| Complete population-based case ascertainment, based on multiple overlapping sources of information? | Yes  No  Unclear |
| Follow-up durations | [months/years] |
| Exclusions |  |
| Data Presentation |  |
| Stroke measurements reported  *Multiple can be selected* | Crude incidence  Age-specific incidence  Attack rates  Cumulative incidence (over time)  Person-years  Other (specify in comments) |
| Complete calendar years of data; not more than 5 years of data averaged together | Yes  No |
| First-ever-in-a-lifetime and recurrent stroke (separately and combined) | Yes  No  Unclear/not specified |
| Follow-up of patients | Yes  No  Unclear/not specified |
| Number of patients evaluated |  |
| Number of Indigenous patients |  |
| Indigenous population denominator |  |
| Number of non-Indigenous patients |  |
| Non-Indigenous population denominator |  |
| Indigenous Age (min); median if no age range |  |
| Indigenous Age (max); leave blank if reporting median |  |
| Non-Indigenous Age (min); median if no age range |  |
| Non-Indigenous Age (max); leave blank if reporting median |  |
| What subgroups were incidence results stratified by or reported for?  *Multiple can be selected* | Male, female  Urban, rural  Age groups  Indigenous status  Sub-type of stroke  Fatal/non-fatal stroke  Other (specify in comments) |
| Type of rate?  *Multiple can be selected* | Crude  Age-adjusted (WHO Standard population)  Age-adjusted (other population)  Crude and age-adjusted (WHO Standard population)  Crude and age-adjusted (other population)  Person-years*  Other (specify in comments) |
| Men and women presented separately? | Yes  No |
| Recommended reporting of age-specific estimates within standard mid-decade age bands? | Yes  No |
| 95% confidence intervals around rates? | Yes  No |
| How were stroke diagnoses validated? | Adjudicated by clinician(s)  Diagnostic imaging reports  Medical notes  ICD codes had been previously validated in that context  ICD codes had been previously validated in similar context  ICD codes unvalidated  Other (specify) |
| How were risk factor data collected? |  |
| Incidence Rates |  |
| **Indigenous** |  |
| Crude |  |
| Age-adjusted (WHO Standard population) |  |
| Age-adjusted (other population) |  |
| Crude and age-adjusted (WHO Standard population) |  |
| Crude and age-adjusted (other population) |  |
| Person-years |  |
| Other (specify in comments) |  |
| *Subgroups (specify below)* |  |
| Male, female |  |
| Urban, rural |  |
| Age groups |  |
| Indigenous status |  |
| Sub-type of stroke |  |
| Mortality |  |
| Fatal/non-fatal stroke |  |
| Other (specify in comments) |  |
| **Non-Indigenous** |  |
| Crude |  |
| Age-adjusted (WHO Standard population) |  |
| Age-adjusted (other population) |  |
| Crude and age-adjusted (WHO Standard population) |  |
| Crude and age-adjusted (other population) |  |
| Person-years |  |
| Other (specify in comments) |  |
| *Subgroups (specify below)* |  |
| Male, female |  |
| Urban, rural |  |
| Age groups |  |
| Indigenous status |  |
| Sub-type of stroke |  |
| Mortality |  |
| Fatal/non-fatal stroke |  |
| Other (specify in comments) |  |
| CONSIDER STATEMENT: Did the study… |  |
| *Governance* |  |
| Describe partnership agreements between the research institution and Indigenous-governing organization for the research? | Yes  No  Unclear |
| Describe accountability and review mechanisms within the partnership agreement that addresses harm minimization? | Yes  No  Unclear |
| Specify how the research partnership agreement includes protection of Indigenous intellectual property and knowledge arising from the research, including financial and intellectual benefits generated? | Yes  No  Unclear |
| *Prioritization* |  |
| Explain how the research aims emerged from priorities identified by either Indigenous stakeholders, governing bodies, funders, non-government organization(s), stakeholders, consumers, and empirical evidence? | Yes  No  Unclear |
| Relationships (Indigenous stakeholders/participants and Research team) | Yes  No  Unclear |
| Specify measures that adhere and honor Indigenous ethical guidelines, processes, and approvals for all relevant Indigenous stakeholders, recognizing that multiple Indigenous partners may be involved? | Yes  No  Unclear |
| Report how Indigenous stakeholders were involved in the research processes? | Yes  No  Unclear |
| Describe the expertise of the research team in Indigenous health and research? | Yes  No  Unclear |
| *Methodologies* |  |
| Describe the methodological approach of the research including a rationale of methods used and implication for Indigenous stakeholders? | Yes  No  Unclear |
| Describe how the research methodology incorporated consideration of the physical, social, economic and cultural environment of the participants and prospective participants. | Yes  No  Unclear |
| *Participation* |  |
| Specify how individual and collective consent was sought to conduct future analysis on collected samples and data. | Yes  No  Unclear |
| Described how the resource demands (current and future) placed on Indigenous participants and communities involved in the research were identified and agreed upon including any resourcing for participation, knowledge, and expertise | Yes  No  Unclear |
| Specify how biological tissue and other samples including data were stored, explaining the processes of removal from traditional lands, if done, and of disposal. | Yes  No  Unclear |
| *Capacity* |  |
| Explain how the research supported the development and maintenance of Indigenous research capacity. | Yes  No  Unclear |
| Discuss how the research team undertook professional development opportunities to develop the capacity to partner with Indigenous stakeholders. | Yes  No  Unclear |
| *Analysis and interpretation* |  |
| Specify how the research analysis and reporting supported critical inquiry and a strength-based approach that was inclusive of Indigenous values. | Yes  No  Unclear |
| *Dissemination* |  |
| Describe the dissemination of the research findings to relevant Indigenous governing bodies and peoples. | Yes  No  Unclear |
| Discuss the process for knowledge translation and implementation to support Indigenous advancement | Yes  No  Unclear |
| Other Comments |  |

* We will convert person-years to X/100,000 per year (acknowledging where this was done)
